# Clinical utility of FDG-PET/CT for post-surgery surveillance of malignant pleural mesothelioma – Comparison with contrast-enhanced CT

**DOI:** 10.18632/oncotarget.27324

**Published:** 2019-11-26

**Authors:** Kazuhiro Kitajima, Masaki Hashimoto, Takayuki Katsuura, Nobuyuki Kondo, Toshiyuki Minami, Kozo Kuribayashi, Seiki Hasegawa, Takashi Kijima, Koichiro Yamakado

**Affiliations:** ^1^Division of Nuclear Medicine and PET Center, Department of Radiology, Hyogo College of Medicine, Nishinomiya, Hyogo, Japan; ^2^Department of Thoracic Surgery, Hyogo College of Medicine, Nishinomiya, Hyogo, Japan; ^3^Division of Respiratory Medicine, Department of Internal Medicine, Hyogo College of Medicine, Nishinomiya, Hyogo, Japan; ^4^Department of Radiology, Hyogo College of Medicine, Nishinomiya, Hyogo, Japan

**Keywords:** mesothelioma, positron emission tomography, computed tomography, recurrence

## Abstract

**Objectives:**

To assess the diagnostic accuracy of fluorodeoxyglucose (FDG)-positron emission tomography/computed tomography (PET/CT) findings for recurrent malignant pleural mesothelioma (MPM) after a radical surgery procedure and their impact on clinical management in comparison with contrast-enhanced CT.

**Results:**

Treatment failure was confirmed in 40 patients. The patient-based area under the receiver-operating characteristic (ROC) curves (AUC)/sensitivity/specificity/accuracy were 0.915/90.0%/80.0%/88.0% for FDG-PET/CT, and 0.805/75.0%/90.0%/78.0% for contrast-enhanced CT, respectively. AUC and sensitivity values were significantly different between the modalities (both p=0.041). Patient-based AUC values for diagnosing locoregional recurrence (ipsilateral hemithoracic recurrence) and distant metastasis, including peritoneal dissemination and lung, bone, muscle, and liver metastasis, were also significantly different (p=0.023 and p=0.035, respectively). The findings of FDG-PET/CT resulted in a change of management for 14 of the 50 patients (28%) by initiating new treatment. Of six patients judged as not having recurrence by contrast-enhanced CT but truly having recurrence based on FDG-PET/CT findings, 4 patients received new treatment due toFDG-PET/CT.

**Methods:**

Fifty patients who underwent radical surgery for MPM received FDG-PET/CT and contrast-enhanced neck/chest/abdomen/pelvis CT examinations for surveillance or suspected recurrence within a 2-week period. Diagnostic ability was determined on a patient and lesion-site basis by 2 experienced examiners, and the modalities were compared using ROC analysis and McNemar test results. Lesion status was determined on the basis of histopathology, radiological imaging and clinical follow-up for longer than 6 months.

**Conclusion:**

FDG-PET/CT findings were shown to be more accurate for assessing MPM recurrence and more often led to therapy change than contrast-enhanced CT.

## INTRODUCTION

Advances in both surgical and nonsurgical therapeutic strategies for cancer continue, cancer recurrence and distant metastasis following the initial treatment remain as major issues for patients with malignant pleural mesothelioma (MPM). Early and accurate detection of recurrence in these patients has been shown to have an important influence on therapy choices, with selection of an appropriate subsequent treatment strategy expected to significantly impact survival [1].

Contrast-enhanced computed tomography (CT) is a widely used modality for assessing treatment failure in MPM patients. Unfortunately, those results are not always specific enough to distinguish between a recurrent tumor and benign post-therapeutic changes. Recent studies have examined use of fluorodeoxyglucose (FDG)-positron emission tomography/computed tomography (PET/CT) for detecting recurrent [2-4]. This imaging modality detects increased utilization of glucose by malignant cells, thus revealing their high glucose uptake, making diagnosis of cancer recurrence and distant metastasis in the preclinical stage possible earlier than with more conventional techniques. As a result, FDG-PET/CT results are considered to have a higher level of accuracy for diagnosis and restaging of MPM than contrast-enhanced CT. In the present study, we assessed FDG-PET/CT regarding its clinical usefulness for diagnosis of recurrent and metastatic MPM, and compared the findings with those obtained using contrast-enhanced CT.

## RESULTS

### Patient-based diagnostic analysis

**Figure 1:**
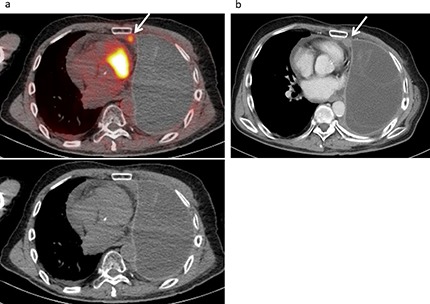
Local recurrence developed in a 67-year-old male following treatment for a malignant pleural mesothelioma, including neoadjuvant chemotherapy, left extrapleural pneumonectomy, radiotherapy, and chemotherapy. **(a)** FDG-PET/CT and CT findings showed low level mild FDG uptake (SUVmax 3.28) in the ipsilateral mediastinal pleura (arrow), suggesting local recurrence with a score of 4. **(b)** Contrast-enhanced CT revealed a tiny enhanced mass (10×11 mm) in the ipsilateral mediastinal pleura (arrow) with a score of 3 (false negative).

**Figure 2:**
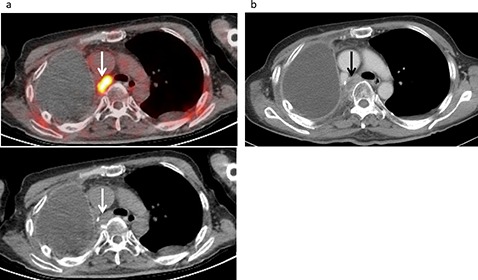
Lymph node recurrence developed in a 61-year-old male following treatment for a malignant pleural mesothelioma, including neoadjuvant chemotherapy, right extrapleural pneumonectomy, and radiotherapy. **(a)** FDG-PET/CT and CT findings showed intense FDG uptake (SUVmax 11.9) in a swollen mediastinal lymph node (arrow), strongly suggesting nodal metastasis with a score of 5. **(b)** Contrast-enhanced CT revealed a swollen mediastinal lymph node (12×18 mm) (arrow), suggesting nodal metastasis with a score of 4.

**Figure 3:**
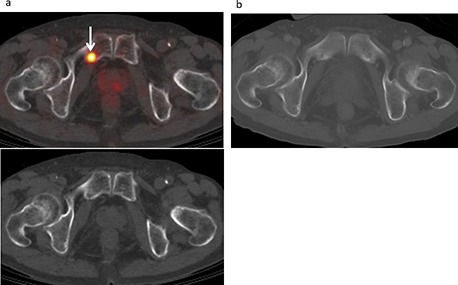
Bone metastasis developed in a 72-year-old male treated for malignant pleural mesothelioma, including pleurectomy and decortication procedures, and chemotherapy. **(a)** FDG-PET/CT and CT findings showed moderate FDG uptake (SUVmax 5.14) in the right pubic bone (arrow), suggesting bone metastasis with a score of 4. **(b)** Contrast-enhanced CT (bone image) results showed no abnormal FDG uptake with a score of 1 (false negative).

**Figure 4:**
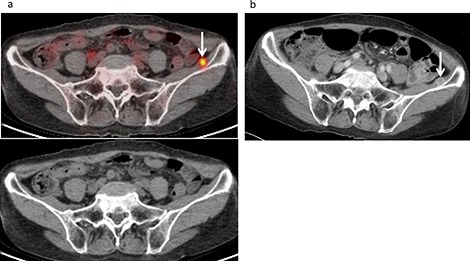
Muscle metastasis developed in a 73-year-old female following treatment for malignant pleural mesothelioma, including neoadjuvant chemotherapy, pleurectomy and decortication procedures, and chemotherapy. **(a)** FDG-PET/CT and CT findings revealed moderate FDG uptake (SUVmax 4.2) in the left iliac muscle (arrow), suggesting muscle metastasis with a score of 4. **(b)** Contrast-enhanced CT revealed an enhanced mass (10 mm) in the left iliac muscle (arrow), which had not been detected by either of the examiners (false negative).

**Figure 5:**
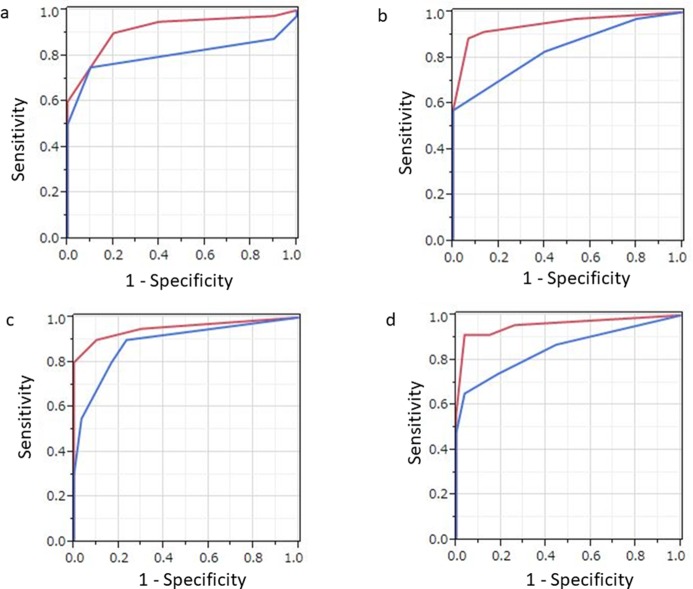
Results of ROC analysis of PET/CT (red) and contrast-enhanced CT (blue) findings regarding. **(a)** whole-lesion involvement, **(b)** thoracic involvement, **(c)** lymph node involvement, and **(d)** distant metastasis in individual patients following surgery for malignant pleural mesothelioma. (a) The PET/CT value for AUC (0.915) in cases with whole-lesion involvement was significantly greater than that (0.805) for contrast-enhanced CT (p=0.041). (b) The PET/CT value for AUC (0.946) in cases with thoracic involvement was significantly greater than that (0.889) for contrast-enhanced CT (p=0.032). (c) The PET/CT value for AUC in cases with nodal involvement tended to be greater than that for contrast-enhanced CT, though the difference was not significant (p=0.1). (d) The PET/CT value for AUC (0.957) in cases with distant metastasis was significantly greater than that (0.852) for contrast-enhanced CT (p=0.035).

Table 1 shows patient demographics and clinicopathologic variables. Forty (80%) of the 50 enrolled patients had confirmed treatment failure. Tumor recurrence confirmation after 2 imaging examinations scans was determined by biopsy (n=10), cytology of ascites (n=2), or average clinical and imaging follow-up (n=28) findings over a period of at least 6 months. Four representative cases are presented in Figures 1–4. Using the FDG-PET/CT results, the value for patient-based AUC was 0.915, and values for sensitivity, specificity, positive predictive value (PPV), negative predictive value (NPV), and accuracy for detecting MPM recurrence were, 90.0% (36/40), 80.0% (8/10), 94.7% (36/38), 66.7% (8/12), and 88.0% (44/50), respectively, while those for contrast-enhanced CT were 0.805, 75.0% (30/40), 90.0% (9/10), 96.8% (30/31), 47.4% (9/19), and 78.0% (39/50), respectively(Table 2) (Figure 5). AUC and sensitivity values with FDG-PET/CT were significantly higher as compared to those with contrast-enhanced CT (p=0.041 and p=0.041, respectively).

**Table 1:** Patient characteristics

	No.	%
Gender		
Male	40	80.0%
Female	10	20.0%
Age, years		
Mean	62.5±8.7	
Range	37-73	
Histological subtype		
Epithelial	46	92.0%
Biphasic	3	6.0%
Small	1	2.0%
Initial clinical staging		
cT1/T2/T3/T4	15/17/16/2	30.0 /34.0 /32.0 /4.0 %
cN0/N1/N2/N3	40/0/10/0	80.0/20.0 %
cM0/M1	50/0	100/0 %
cStageI/ II/III/IV	14/18/16/2	28.0/36.0/32.0/4.0 %
Initial pathological staging		
pT1/T2/T3/T4	11/13/24/2	22.0/26.0/48.0/4.0 %
pN0/N1/N2/N3	39/0/11/0	78.0/0/22.0/0 %
pM0/M1	50/0	100/0 %
pStageI/ II/III/IV	10/11/27/2	20.0/22.0/34.0/4.0 %
Surgery type		
Extrapleural pneumonectomy (EPP)	29	58.0%
Pleurectomy/decortication (P/D)	21	42.0%
Previous therapy		
Neoadjuvant chemotherapy+EPP	6	12.0%
Neoadjuvant chemotherapy+P/D	1	2.0%
Neoadjuvant chemotherapy+EPP+RT	11	22.0%
Neoadjuvant chemotherapy+P/D+RT	2	4.0%
Neoadjuvant chemotherapy+EPP+chemotherapy	2	4.0%
Neoadjuvant chemotherapy+P/D+chemotherapy	4	8.0%
Neoadjuvant chemotherapy+EPP+RT+chemotherapy	10	20.0%
Neoadjuvant chemotherapy+P/D+RT+hemotherapy	14	28.0%

No: number, RT: radiotherapy, EPP: extrapleural pneumonectomy, P/D: pleurectomy and decortication

**Table 2:** Comparison of patient-based diagnostic performance for diagnosing MPM recurrence between FDG-PET/CT and contrast-enhanced CT

	AUC	Sensitivity	Specificity	PPV	NPV	Accuracy
	95%CI	95%CI	95%CI	95%CI	95%CI	95%CI
Whole-lesion						
PET/CT	0.915	90.0% (36/40)	80.0% (8/10)	94.7% (36/38)	66.7% (8/12)	88.0% (44/50)
	0.795-0.968	80.7-99.3	55.2-100	87.6-100	40.0-93.3	79.0-97.0
CECT	0.805	75.0% (30/40)	90.0% (9/10)	96.8% (30/31)	47.4% (9/19)	78.0% (39/50)
	0.668-0.895	61.6-88.4	71.4-100	90.5-100	24.9-69.8	66.5-89.5
p value	0.041	0.041	1.0			0.074
Thoracic recurrence						
PET/CT	0.946	91.4% (32/35)	86.7% (13/15)	94.1% (32/34)	81.3% (13/16)	90.0% (45/50)
	0.847-0.982	80.7-99.3	55.2-100	86.2-100	62.1-100	81.7-98.3
CECT	0.837	82.9% (29/35)	60.0% (9/15)	82.9% (29/35)	60.0% (9/15)	76.0% (38/50)
	0.709-0.915	70.4-95.3	35.2-84.8	70.4-95.3	35.2-84.8	64.2-87.8
p value	0.032	0.25	0.13			0.023
Lymph node involvement						
PET/CT	0.953	80.0% (16/20)	100% (30/30)	100% (16/16)	88.2% (30/34)	92.0% (46/50)
	0.821-0.989	62.5-97.5	100	87.6-100	77.4-99.1	84.5-99.5
CECT	0.889	55.0% (11/20)	96.7% (29/30)	91.7% (11/12)	76.3% (29/38)	80.0% (40/50)
	0.757-0.954	33.2-76.8	90.2-100	84.0-99.3	62.8-89.8	68.9-91.1
p value	0.1	0.074	1.0			0.041
Distant metastasis						
PET/CT	0.957	91.3% (21/23)	85.2% (23/27)	84.0% (21/25)	92.0% (23/25)	88.0% (44/50)
	0.844-0.989	79.8-100	71.8-98.6	69.6-98.4	81.4-100	79.0-97.0
CECT	0.852	73.9% (17/23)	81.5% (22/27)	77.3% (17/22)	78.6% (22/28)	78.0% (39/50)
	0.706-0.932	56.0-91.9	66.8-96.1	59.8-94.8	63.4-93.8	66.5-89.5
p value	0.035	0.13	1.0			0.074

AUC: area under receiver-operating characteristic curve, CI: confidence interval, PET/CT: positron emission tomography/computed tomography, CECT: contrast-enhanced computed tomography

Based on FDG-PET/CT findings, there were 4 false-negative and 2 false-positive cases. As for the false-negative cases, there was 1 each of tiny contralateral pleural dissemination, tiny peritoneal dissemination, tiny ipsilateral pleural dissemination and lymph node metastasis, and tiny ipsilateral pleural dissemination and peritoneal dissemination, while the false-positive cases included 1 for which FDG-PET/CT showed increased FDG uptake (SUVmax 6.04) in the right cardiophrenic angle that was misjudged as ipsilateral pleural dissemination (locoregional recurrence) and 1 with a surgery-proven peritoneal desmoid with moderate FDG uptake (SUVmax 3.89) that was misjudged as peritoneal dissemination in FDG-PET/CT findings.

### Lesion site-based diagnostic analysis

Ipsilateral hemithorax (locoregional recurrence), contralateral hemithorax, lymph node, peritoneum, lung, bone, muscle, and liver involvement were documented in 33 (66.0%), 7 (14.0%), 20 (40.0%), 15 (30.0%), 5 (10.0%), 5 (10.0%), 4 (8.0%), and 1 (2.0%), respectively, of the 50 patients (Table 3). Results of analyses of the diagnostic performance of FDG-PET/CT and contrast-enhanced CT (patient-based AUC, sensitivity, specificity, PPV, NPV, accuracy) for those 8 lesion sites are also presented in Table 3.

**Table 3:** Comparison of diagnostic performance for diagnosing MPM recurrence in 8 different lesion sites between FDG-PET/CT and contrast-enhanced CT

	AUC	Sensitivity	Specificity	PPV	NPV	Accuracy
	95%CI	95%CI	95%CI	95%CI	95%CI	95%CI
Locoregional recurrence (ipsilateral hemithoracic recurrence)						
PET/CT	0.958	90.9% (30/33)	94.1% (16/17)	93.8% (30/32)	83.3% (15/18)	90.0% (45/50)
	0.878-0.986	81.1-100	82.9-100	85.4-100	66.1-100	81.7-98.3
CECT	0.838	57.6% (19/33)	100% (17/17)	100% (19/19)	54.8% (17/31)	72.0% (36/50)
	0.714-0.915	40.7-74.4	100	100	37.3-72.4	59.9-84.4
p value	0.023	0.026	1.0			0.0077
Contralateral hemithoracic recurrence						
PET/CT	0.922	85.7% (6/7)	100% (43/43)	100% (6/6)	97.7% (43/44)	98.0% (49/50)
	0.585-0.990	81.1-100	72.9-100	100	93.3-100	94.1-100
CECT	0.897	28.6% (2/7)	97.7% (42/43)	28.6% (2/7)	97.7% (42/43)	88.0% (44/50)
	0.640-0.977	81.1-100	93.2-100	81.1-100	93.2-100	79.0-97.0
p value	0.235	0.13	1.0			0.074
Lymph node involvement						
PET/CT	0.953	80.0% (16/20)	100% (30/30)	100% (16/16)	88.2% (30/34)	92.0% (46/50)
	0.821-0.989	62.5-97.5	100	87.6-100	77.4-99.1	84.5-99.5
CECT	0.889	55.0% (11/20)	96.7% (29/30)	91.7% (11/12)	76.3% (29/38)	80.0% (40/50)
	0.757-0.954	33.2-76.8	90.2-100	84.0-99.3	62.8-89.8	68.9-91.1
p value	0.1	0.074	1.0			0.041
Peritoneal dissemination						
PET/CT	0.922	80.0% (12/15)	97.1% (34/35)	93.8% (12/13)	91.9% (34/37)	92.0% (46/50)
	0.761-0.978	59.8-100	91.6-100	77.8-100	83.1-100	84.5-99.5
CECT	0.916	66.7% (10/15)	97.1% (34/35)	90.9% (10/11)	87.2% (34/39)	88.0% (44/50)
	0.760-0.974	42.8-90.5	91.6-100	73.9-100	76.7-97.7	79.0-97.0
p value	0.53	0.48	1.0			0.48
Lung metastasis						
PET/CT	1.0	100% (5/5)	100% (45/45)	100% (5/5)	100% (45/45)	100% (50/50)
	1.0	100	100	100	100	100
CECT	0.993	80.0% (4/5)	100% (45/45)	100% (4/4)	97.8% (45/46)	98.0% (49/50)
	0.939-0.999	44.9-100	100	100	93.6-100	94.1-100
p value	0.38	1.0	1.0			1.0
Bone metastasis						
PET/CT	1.0	100% (5/5)	100% (45/45)	100% (5/5)	100% (45/45)	100% (50/50)
	1.0	100	100	100	100	100
CECT	0.849	20.0% (1/5)	100% (45/45)	100% (1/1)	91.8% (45/49)	92.0% (46/50)
	0.533-0.965	0-55.1	100	100	84.2-99.5	84.5-99.5
p value	0.15	0.13	1.0			0.13
Muscle metastasis						
PET/CT	1.0	100% (4/4)	100% (46/46)	100% (4/4)	100% (46/46)	100% (50/50)
	1.0	100	100	100	100	100
CECT	0.848	50.0% (2/4)	97.8% (45/46)	66.7% (2/3)	95.7% (45/47)	94.0% (47/50)
	0.442-0.975	1-99.0	93.6-100	13.3-100	87.4-100	84.5-99.5
p value	0.24	0.48	1.0			0.25
Liver metastasis						
PET/CT	1.0	100% (1/1)	100% (49/49)	100% (1/1)	100% (49/49)	100% (50/50)
	1.0	100	100	100	100	100
CECT	1.0	100% (1/1)	100% (49/49)	100% (1/1)	100% (49/49)	100% (50/50)
	1.0	100	100	100	100	100
p value	1.0	1.0	1.0			1.0

AUC: area under receiver-operating characteristic curve, CI: confidence interval, PET/CT: positron emission tomography/computed tomography, CECT: contrast-enhanced computed tomography

The values for AUC, sensitivity, and accuracy of FDG-PET/CT for diagnosing ipsilateral hemithoracic recurrence (locoregional recurrence) were significantly higher as compared to those for contrast-enhanced CT (p=0.023, p=0.026, and p=0.0077, respectively). Additionally, the accuracy of FDG-PET/CT for diagnosing lymph node involvement was significantly higher (p=0.041). As for sensitivity, that of FDG-PET/CT for diagnosing bone and muscle metastasis tended to be higher than that of contrast-enhanced CT, though the difference was not significant.

The mean maximum standardized uptake (SUVmax) value for ipsilateral hemithoracic involvement (locoregional recurrence) in the 33 affected patients was 6.86±4.01 (range 1.4-18.38), while that for contralateral hemithoracic involvement in the 7 affected patients was 4.66±1.65 (range 1.8-6.11). A total of 23 metastatic lymph nodes (12 mediastinal, 3 hilar, 3 abdominal, 3 supraclavicular, 1 inguinal, 1 retrocrural space) were observed in 20 patients, with a mean SUVmax value of 5.16±2.81 (range 0-11.9). The mean SUVmax values for peritoneal dissemination in 15 and for lung metastasis in 5 affected patients were 5.76±4.21 (range 0-12.76) and 3.60±2.71 (range 0-7.27), respectively. In 5 patients, a total of 12 metastatic bone lesions were seen, including rib (n=2), lumbar spine (n=2), sacrum (n=2), iliac bone (n=2), thoracic spine (n=1), pubic bone (n=1), sternum (n=1), and ischium (n=1) locations. The mean SUVmax value for those 12 lesions was 6.49±3.04 (range 2.65-12.29). Furthermore, there were 5 different muscle tissue locations in 4 patients with metastasis (erector spinae, subscapularis, latissimus dorsi, iliac, iliopsoas), with the mean SUVmax for all 9.20±4.91 (range 4.2-15.16). Also, 1 patient had multiple liver metastasis sites, with the highest SUVmax value among them 11.28.

Recurrent lesions were classified as thoracic involvement (ipsilateral and contralateral hemithoracic involvement), nodal involvement, and distant metastasis (peritoneal dissemination, lung metastasis, bone metastasis, muscle metastasis, liver metastasis) (Table 2) (Figure 5). The patient-based AUC and accuracy values of FDG-PET/CT for detecting thoracic involvement were significantly higher as compared to those of contrast-enhanced CT (p=0.032 and p=0.023, respectively). Additionally, patient-based accuracy for detecting lymph node metastasis and patient-based AUC for detecting distant metastasis of FDG-PET/CT were significantly higher as compared to those of contrast-enhanced CT (p=0.035).

“EPP vs P/D

Patient-based performance for diagnosing MPM recurrence in patients undergoing EPP (n=29) and P/D (n=21) are compared in Table 4. In a EPP group (n=29), patient-based values for AUC, sensitivity, specificity, PPV, NPV, and accuracy for detection of whole lesion were 0.873, 91.3% (21/23), 66.7% (4/6), 91.3% (21/23), 66.7% (4/6), and 86.2% (25/29), respectively, for FDG-PET/CT, and 0.732, 69.6% (16/23), 83.3% (5/6), 94.1% (16/17), 41.7% (5/12), and 72.4% (21/29), respectively, for contrast-enhanced CT. On the other hand, in a P/D group (n=21) , patient-based values for AUC, sensitivity, specificity, PPV, NPV, and accuracy for detection of whole lesion were 0.963, 88.2% (15/17), 100% (4/4), 100% (15/15), 66.7% (4/6), and 90.5% (19/21), respectively, for FDG-PET/CT, and 0.902, 82.4% (14/17), 100% (4/4), 100% (14/14), 57.1% (4/7), and 85.7% (18/21), respectively, for contrast-enhanced CT. All sensitivity, accuracy, and AUC of FDG-PET/CT were higher than those of contrast-enhanced CT in both EPP and P/D group without significant difference.

**Table 4:** Patient-based performance for diagnosing MPM recurrence between FDG-PET/CT and contrast-enhanced CT according to the surgical type

		AUC	Sensitivity	Specificity	PPV	NPV	Accuracy
Whole-lesion							
EPP (n=29)	PET/CT	0.873	91.3% (21/23)	66.7% (4/6)	91.3% (21/23)	66.7% (4/6)	86.2% (25/29)
	CECT	0.732	69.6% (16/23)	83.3% (5/6)	94.1% (16/17)	41.7% (5/12)	72.4% (21/29)
	p value	0.051	0.074	1			0.13
P/D (n=21)	PET/CT	0.963	88.2% (15/17)	100% (4/4)	100% (15/15)	66.7% (4/6)	90.5% (19/21)
	CECT	0.902	82.4% (14/17)	100% (4/4)	100% (14/14)	57.1% (4/7)	85.7% (18/21)
	p value	0.38	1	1			1
Thoracic recurrence							
EPP (n=29)	PET/CT	0.919	88.9% (16/18)	90.9% (10/11)	94.1% (16/17)	83.3% (10/12)	89.7% (26/29)
	CECT	0.763	77.8% (14/18)	63.6% (7/11)	77.8% (14/18)	63.6% (7/11)	72.4% (21/29)
	p value	0.032	0.48	0.25			0.074
P/D (n=21)	PET/CT	0.941	94.1% (16/17)	75.0% (3/4)	94.1% (16/17)	75.0% (3/4)	90.5% (19/21)
	CECT	0.854	88.2% (15/17)	50.0% (2/4)			81.0% (17/21)
	p value	0.21	1	1			0.48
Lymph node involvement							
EPP (n=29)	PET/CT	0.934	81.8% (9/11)	100% (18/18)	100% (9/9)	95.0% (18/20)	93.1% (27/29)
	CECT	0.864	54.5% (6/11)	100% (18/18)	100% (6/6)	78.3% (18/23)	82.8% (24/29)
	p value	0.19	0.25	1			0.25
P/D (n=21)	PET/CT	0.977	77.8% (7/9)	100% (12/12)	100% (7/7)	85.7% (12/14)	90.5% (19/21)
	CECT	0.884	55.6% (5/9)	91.7% (11/12)	83.3% (5/6)	73.3% (11/15)	76.2% (16/21)
	p value	0.15	0.48	1			0.25
Distant metastasis							
EPP (n=29)	PET/CT	0.931	92.9% (13/14)	86.7% (13/15)	92.9% (13/14)	93.3% (14/15)	89.7% (26/29)
	CECT	0.862	78.6% (11/14)	86.7% (13/15)	84.6% (11/13)	81.3% (13/16)	82.8% (24/29)
	p value	0.23	0.48	1			0.48
P/D (n=21)	PET/CT	0.868	88.9% (8/9)	91.7% (10/12)	100% (8/8)	92.3% (12/13)	85.7% (18/21)
	CECT	0.752	66.7% (6/9)	75.0% (9/12)	66.7% (6/9)	75.0% (9/12)	71.4% (15/21)
	p value	0.16	0.48	1			0.25

AUC: area under the receiver-operating characteristic curves, EPP: extrapleural pneumonectomy, P/D: pleurectomy and decortication, PET/CT:positron emission tomography/computed tomography, CECT: contrast-enhanced computed tomograpy

Patient-based diagnostic performance for thoratic recurrence, lymph node involvement, and distant metastasis showed similar tendency. The only AUC for diagnosing thoratic recurrence in a EPP group reached the significant difference between FDG-PET/CT and contrast-enhanced CT (p=0.031).”

### Impact on patient management

FDG-PET/CT findings impacted the management of 14 (28.0%) of the 50 patients, including initiation of chemotherapy in 8, radiotherapy in 3, resection in 2, and radiofrequency ablation in 1 as a new treatment modality. Six patients judged to not have recurrence based on contrast-enhanced CT findings were shown to have recurrence in FDG-PET/CT findings, of whom 2 had locoregional recurrence (ipsilateral hemithoracic recurrence), 1 bone metastasis, 1 muscle metastasis, 1 nodal involvement, and 1 lung and bone metastasis, and locoregional recurrence (ipsilateral hemithoracic recurrence). Two of those patients underwent sequential chemotherapy treatments, thus FDG-PET/CT was considered to have had an influence on additional therapeutic management (chemotherapy) in 4 cases.

## DISCUSSION AND CONCLUSION

Using FDG-PET/CT findings, the patient-based value for AUC was 0.915, while those values for sensitivity, specificity, PPV, NPV, and accuracy for detecting MPM recurrence were, 90.0%, 80.0%, 94.7%, 66.7%, and 88.0%, respectively. As for contrast-enhanced CT, the values were 0.805, 75.0%, 90.0%, 96.8%, 47.4%, and 78.0%, respectively. Thus, both AUC and sensitivity for FDG-PET/CT were significantly greater (p=0.041 and p=0.041, respectively). In addition, patient-based AUC values for diagnosis of locoregional recurrence (ipsilateral hemithoracic recurrence) and distant metastasis, including peritoneal dissemination and lung, bone, muscle, and liver metastasis, were significantly different between these imaging modalities (p=0.032 and p=0.035, respectively).

Previous studies evaluated FDG-PET/CT diagnostic performance for detection of MPM recurrence following a radical surgery procedure, including extrapleural pneumonectomy (EPP) and pleurectomy/decortication (P/D) [2-4]. Those authors reported patient-based sensitivity, specificity, PPV, NPV, and accuracy values of FDG-PET/CT for detecting MPM recurrence following radical surgery ranging from 94-98%, 75-100%, 86-100%, 83-95%, and 94-98%, respectively, similar to those found in the present cohort. In an investigation that included 57 patients, of whom 35 underwent surgery, Niccoli-Asabella et al. [4] noted those patient-based values for detecting MPM recurrence after various treatments as 98.1%, 100%, 100%, 83.3%, and 98.2%, respectively, for FDG-PET/CT, and 96.2%, 20.0%, 92.5%, 33.3%, and 89.4%, respectively, for contrast-enhanced CT. Again, the present findings were similar and demonstrate that FDG-PET/CT is more accurate for diagnosis of MPM recurrence in patients who have undergone radical surgery as compared to contrast-enhanced CT. As for our cohort, FDG-PET/CT findings had an impact on management, as they indicated the need for new treatment in 14 (35%) of 40 patients with confirmed failure. Also, Gerbaudo et al. [3] reported that FDG-PET/CT findings were helpful to select 12 (29%) of their 42 patients for an additional previously unplanned treatment because of evidence of initial treatment failure.

To our knowledge this is the first study to compare the diagnostic value of FDG-PET/CT and contrast-enhanced CT between EPP and P/D groups separately. A similar tendency was observed in between in EPP and P/D groups and the only AUC for diagnosing thoratic recurrence in a EPP group reached the significant difference between FDG-PET/CT and contrast-enhanced CT. Although FDG-PET/CT may be more valuable to detect thoratic recurrence after EPP than after PD, this should be confirmed in a prospective and multicenter study with large number of patients.”

The present study has some limitations, including the relatively few patients, who were enrolled from a single institution. There also may have been bias regarding patient selection because of the retrospective design and strict criteria for patient enrollment. The gold standard for analysis of this type is histological confirmation of results. Nevertheless, clinical follow-up examination results are considered valid for diagnostic accuracy and therapy response evaluations, and it would have been unethical to utilize an invasive procedure to investigate all PET/CT-detected lesions. Finally, a consensus method for determining results, as employed in this study, is not preferred, with the ideal being independent determinations with formal interobserver variability.

In conclusion, the present results showed FDG-PET/CT superior for assessment of MPM recurrence. Additionally, those findings led to a change in subsequent appropriate therapy more often than findings obtained with contrast-enhanced CT.

## MATERIALS AND METHODS

### Patients

From May 2006 to December 2017, 149 patients underwent a radical surgery procedure at our institution for histopathologically proven MPM, including EPP and P/D. Thereafter, 131 were regularly followed on an outpatient basis and received contrast-enhanced CT examinations for surveillance, which included 110 who also received whole-body FDG-PET/CT examinations. Following surgery, most of the patients received a contrast-enhanced CT examination every 3 months and FDG-PET/CT examination every 6 or 12 months for surveillance. For the present study, 50 patients (40 males, 10 females; average 62.5 years old, range 37-73 years old) who had received both whole-body FDG-PET/CT and contrast-enhanced neck/chest/abdomen/pelvis CT examinations for surveillance or suspected recurrence within 2 weeks were enrolled. Of those, 31 underwent contrast-enhanced CT first, followed by FDG-PET/CT, while 19 underwent FDG-PET/CT and then contrast-enhanced CT. The mean interval between surgery and the initial imaging scan (FDG-PET/CT or contrast-enhanced CT) was 416.3±115.6 days (range 169-612 days). Tumor recurrence confirmation after the 2 imaging examinations was determined from histologic or cytologic analysis findings, or after at least 6 months of clinical and imaging follow-up examinations, such as contrast-enhanced CT, FDG-PET/CT, and/or magnetic resonance imaging (MRI). Table 1 shows patient demographics and clinicopathologic variables.

### FDG-PET/CT and contrast-enhanced CT examinations

The FDG-PET/CT examinations were performed using a PET/CT scanner (Gemini GXL16 or Gemini TF64; Philips Medical Systems, Eindhoven, The Netherlands) equipped with a gadolinium oxyorthosilicate detector. Details of the FDG-PET/CT procedures have been described [5]. Briefly, patients were instructed to fast for 5 hours before the scan, then blood glucose was measured immediately prior to injection of FDG at 4.0 MBq/kg body weight for the GXL16 and 3.0 MBq/kg for the TF64. All in the present cohort had a blood glucose level lower than 160 mg/dL. Approximately 60 minutes after the injection, static emission images were obtained. Helical CT scan images from the top of the head to the bottom of the feet were obtained for attenuation correction and anatomic localization, using the following parameters: tube voltage 120 kV, effective tube current auto-mA up to 120 mA (GXL16) or 100 mA (TF64), gantry rotation speed 0.5 seconds, detector configuration 16×1.5 mm (GXL16) or 64×0.625 mm (TF64), slice thickness 2 mm, and transverse field of view 600 mm. Immediately upon completion of the CT examination, PET images from the head to the mid-thigh were acquired for 90 seconds per bed position and from the mid-thigh to the toes for 30 seconds per bed position using a variable sampling method. Next, images at 12-14 bed positions for 90 seconds each and 6-7 bed positions for 30 seconds each were obtained in 3-dimensional mode. Thus, 22 to 24-26 minutes of emission scanning was required for each patient. During PET scanning, the patients were allowed to breathe normally.

Pre-contrast and contrast-enhanced CT images of the neck, chest, abdomen, and pelvis were obtained using a 128-detector row CT (SOMATOM Definition AS) at 120 kV, with an effective mA of 220 (CAREDose4D), beam pitch of 0.6, collimation of 1.2×32 mm, and B31 + medium smooth + image reconstruction. The detailed procedures for contrast-enhanced CT have been described [5]. Briefly, the level of blood creatinine was checked prior to the examination and did not exceed 1.5 mg/dL in any of the patients. Using a power injector, iodinated contrast material (Iopamiron Inj, Syringe, Bayer Schering Pharma, Berlin, Germany) containing 300 mg of iodine per ml at a dose of 600 mg of iodine per kg of body weight was intravenously administered, then scanning was started at 120 seconds after the injection.

### Image analysis

FDG-PET/CT images were interpreted on a retrospective basis and consensus determinations made by 2 board-certified nuclear medicine physician and radiologists with 10 and 5 years of experience, respectively, in FDG-PET/CT, and no knowledge of the other imaging results or clinical data for the present cohort. A recurrent or metastatic lesion was diagnosed when abnormal focal FDG uptake shown by PET corresponded to an abnormal mass revealed by CT. Lymph nodes with increased glucose uptake were considered positive for metastatic spread, even in cases when the short-axis diameter was less than 1 cm [6], whereas cases with no detectable tracer uptake were considered negative for metastatic spread even when the short-axis diameter was 1 cm or greater.

Semiquantitative analysis of abnormal radiotracer uptake for each lesion was also performed using SUVmax values, calculated as follows: SUV = volume of interest radioactivity concentration (Bq/mL)/[injected dose (Bq)/patient weight (g)]. SUVmax was defined as the greatest SUV value for pixels with the highest count within the VOI, with the highest for each used as the representative value of each scan. GI-PET (AZE Co., Ltd., Tokyo, Japan), a commercially available software package able to harmonize SUV values across different PET/CT systems [7], was employed.

Contrast-enhanced CT images were retrospectively evaluated and consensus determinations made by 2 board-certified radiologist and nuclear medicine physicians, with 17 and 10 years of experience, respectively, with CT, and no knowledge of the other imaging results or clinical data used in the present study. Images of coronal, axial, and sagittal sections were viewed and analyzed, with appropriate winding applied. A lymph node with a short-axis diameter greater than 1 cm was defined as malignant. A central unenhanced area suggesting central necrosis was also considered to be a sign of malignancy, whereas the presence of peripheral low attenuation suggesting a fatty hilum within a lymph node was considered to indicate benign, regardless of node size [6].

The diagnostic ability of the scanning modalities was determined for each individual patient as well as 8 different lesion sites, including ipsilateral hemithorax (locoregional recurrence), contralateral hemithorax, lymph node, peritoneum, lung, bone, muscle, and liver, with a 5-point grading system (1, definitely absent; 2, probably absent; 3, indeterminate; 4, probably present; 5, definitely present) utilized [8]. Each set of data was reviewed in random order after an interval of at least 4 weeks to avoid decision threshold bias.

### Statistical analysis

Patient- and lesion site-based analyses of the PET/CT results obtained by consensus in comparison with contrast-enhanced CT findings were performed. PET/CT and contrast-enhanced CT results for each region were also compared with the true lesion status (gold standard), then classified as true positive, false positive, true negative, or false negative. Using standard statistical formulae, we then determined sensitivity, specificity, PPV, NPV, and accuracy, with the 95% confidence interval (95% CI) value calculated for each parameter. For sensitivity and specificity, a score of 4 of 5 was considered to indicate positive, because of higher diagnostic accuracy. A McNemar test was used for differences between the 2 imaging modalities, with a *p* value less than 0.05 considered to indicate statistical significance. All analyses were performed using the SAS software package, version 9.3 (SAS Institute, Cary, NC).

## Abbreviations

FDG: fluorodeoxyglucose
PET/CT: positron emission tomography/computed tomography
MPM: malignant pleural mesothelioma
ROC: receiver-operating characteristic
AUC: area under the receiver-operating characteristic curves
CT: contrast-enhanced computed tomography
PPV: positive predictive value
NPV: negative predictive value
SUVmax: maximum standardized uptake
EPP: extrapleural pneumonectomy
P/D: pleurectomy/decortication
